# Surface Display of an Anti-CD3 scFv by *Saccharomyces boulardii* and Its Effect on DSS-Induced Colitis in Mice

**DOI:** 10.3390/ijms27208959

**Published:** 2026-10-09

**Authors:** Sylvia Barbosa Pinhate, Pietra Oliveira Guimarães, Isabel Garcia Sousa, Manuela Maragno do Almo, Renato Rodrigues Oliveira, Ana Luiza Tancredi de Matos, Andréa Queiroz Maranhão, Marcelo Macedo Brigido

**Affiliations:** 1 Molecular Immunology Group, Department of Cell Biology, University of Brasília, Brasília 70910-900, DF, Brazil; sylviapinhate@gmail.com (S.B.P.); pietraoliveirag@gmail.com (P.O.G.); isagarsousa@gmail.com (I.G.S.); manu.maragno5@gmail.com (M.M.d.A.); renatoliver201@gmail.com (R.R.O.); analuizatancredi@gmail.com (A.L.T.d.M.); andreaqm@unb.br (A.Q.M.); 2 Molecular Biology Graduate Program, Biology Institute, University of Brasília, Brasília 70910-900, DF, Brazil; 3 Molecular Pathology Graduate Program, Faculty of Medicine, University of Brasília, Brasília 70910-900, DF, Brazil; 4 National Institute of Science and Technology of Mucosa and Skin, Belo Horizonte 31270-901, MG, Brazil

**Keywords:** anti-CD3, colitis, heterologous gene expression, probiotic yeast, *Saccharomyces boulardii*, surface display

## Abstract

*Saccharomyces boulardii* (Sb) is a probiotic yeast amenable to genetic manipulation, making it a promising platform for delivery of recombinant biotherapeutics to the intestinal lumen. In this study, we engineered a recombinant Sb strain to display an anti-CD3 single-chain variable fragment (scFv) on its surface and evaluated its potential as an oral antibody delivery system. To improve antibody accessibility, we tested different stalk lengths between the scFv and the SED1 anchoring domain. The strain with the longer stalk domain (Sb649) showed more stable surface display over time and was subsequently evaluated in a dextran sulfate sodium (DSS)-induced colitis model. Sb649-treated mice showed body weight values and disease activity profiles closer to those of healthy controls. Sb649 administration was also associated with transcriptional changes, including increased *Foxp3* expression and no increase in *Il6* in mesenteric lymph nodes, unlike DSS and DSS+SbWT-treated mice, along with increased *Muc3* expression in the colon compared to DSS-treated mice. Together, these findings show that engineered Sb can stably display antibody fragments and support its potential as a platform for oral delivery of antibody-based immunotherapies to the gastrointestinal tract.

## 1. Introduction

Antibody-based therapies have become increasingly important in modern medicine, as monoclonal antibodies (mAbs) can target therapeutically relevant molecules, such as pro-inflammatory cytokines [1]. However, some mAbs, such as anti-CD3, can bind to surface receptors, induce tolerance, and stimulate T regulatory cells (Tregs) [2]. Inflammatory bowel diseases (IBD), which include ulcerative colitis (UC) and Crohn’s disease (CD), involve dysregulated inflammatory responses in the gastrointestinal tract (GIT). In UC, inflammation of the colonic and rectal mucosa results from intestinal barrier disruption, which drives an exacerbated immune response, changes in microbiota, and tissue injury [3]. Additionally, increased activation and proliferation of CD4^+^ T-cells promote pro-inflammatory cytokine production and continuous inflammatory stimuli [4,5]. In the GIT, anti-CD3 can reduce colonic inflammation by attenuating CD4^+^ effector T-cell responses [6].

Due to its role in disease development and pathogenesis, the gut microbiota is a key therapeutic target in UC [7]. Probiotics can help control UC symptoms, maintain remission, promote a balanced microbiota, and strengthen the intestinal barrier [8,9]. Therefore, genetically modified probiotics have emerged as a promising strategy for the localized production and delivery of therapeutic molecules in the gut while also addressing dysbiosis [10]. Additionally, increased cell recruitment and disruption of the epithelial barrier in the inflamed gut may allow more probiotic-immune system interactions and amplify beneficial effects [11]. Therefore, identifying suitable microorganisms for this biotechnological approach is necessary.

Among the available candidates, *Saccharomyces boulardii* (Sb) stands out. This probiotic yeast was first isolated from lychees by Henri Boulard in 1920 [12,13], is generally recognized as safe (GRAS) by the Food and Drug Administration, and is used to treat and prevent intestinal disorders [14]. In animal colitis models, its use maintained epithelial integrity, reduced pro-inflammatory NF-κB signaling, and stimulated the Nrf2-mediated antioxidant response [15]. In addition, *S. boulardii* does not permanently colonize the gut and can efficiently express recombinant proteins in vivo, making it a promising platform for the localized delivery of therapeutic molecules [16,17,18].

Here, we investigated the potential of *S. boulardii* as an immunotherapeutic delivery platform by assessing its ability to express and display an anti-CD3 single-chain variable fragment (scFv) anchored to the cell wall to target T-cells in the gut. Specifically, we investigated scFv surface accessibility and the effects of the engineered yeast in a dextran sulfate sodium (DSS)-induced colitis model.

## 2. Results

### 2.1. Surface Display of Anti-CD3 scFv on the Yeast Cell Wall

**Figure 1:**
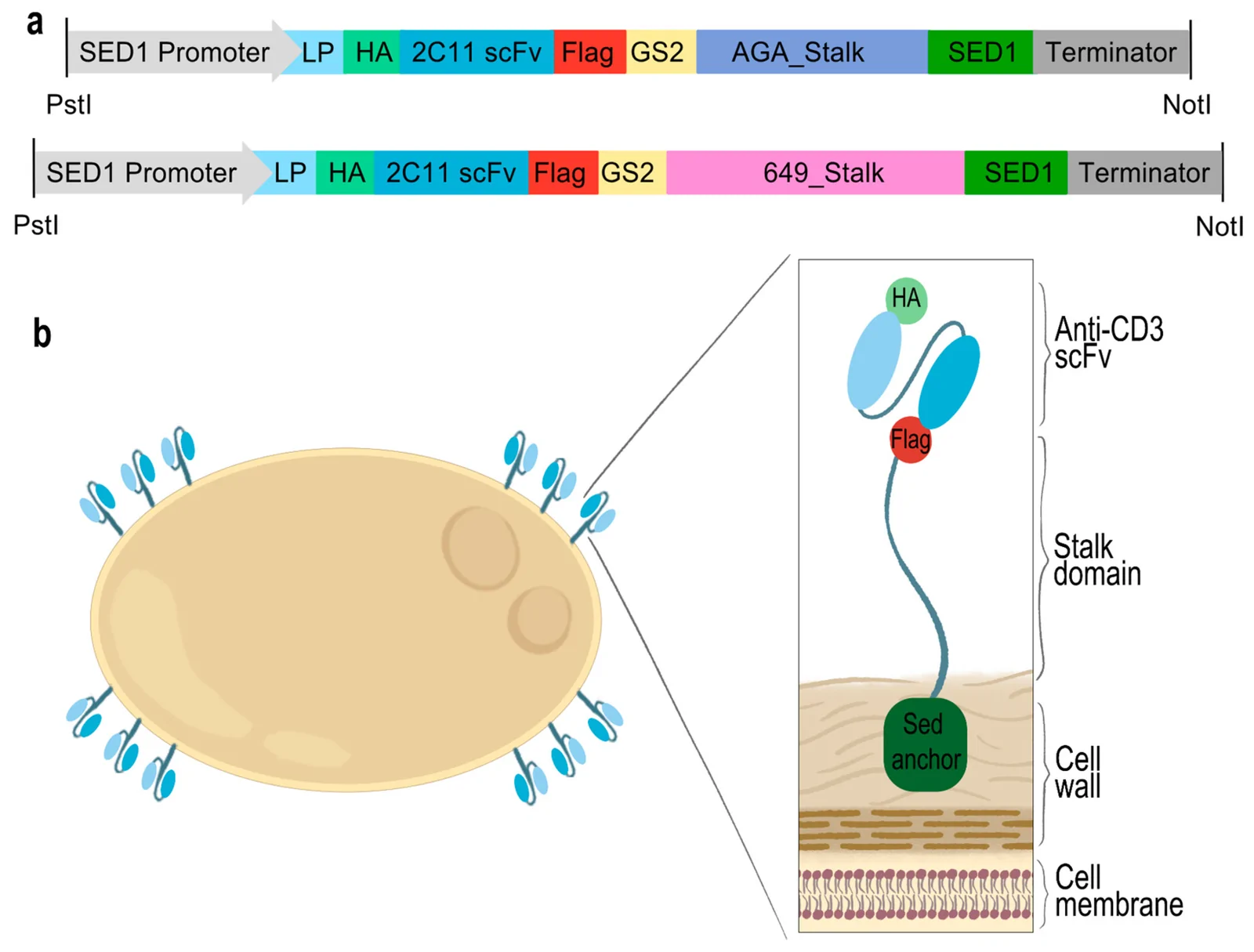
Diagram of the vectors and expression cassettes. (**a**) Schematic representation of the expression cassettes, including the SED1 promoter, leader peptide (LP), murine anti-CD3 scFv (2C11), hemagglutinin (HA) and FLAG tags for protein detection, carboxy-terminal domain, and transcription terminator. The constructs differ in the sequence and length of their stalk domain. (**b**) Schematic representation of cell wall-anchored scFv display in *Saccharomyces boulardii*.

We compared two expression vectors encoding an anti-CD3 scFv fused to a C-terminal cell-wall anchor for surface display. The constructs differed in stalk-domain sequence and length: AGA_Stalk (289 amino acids) and 649_Stalk (649 amino acids) (Figure 1).

**Figure 2:**
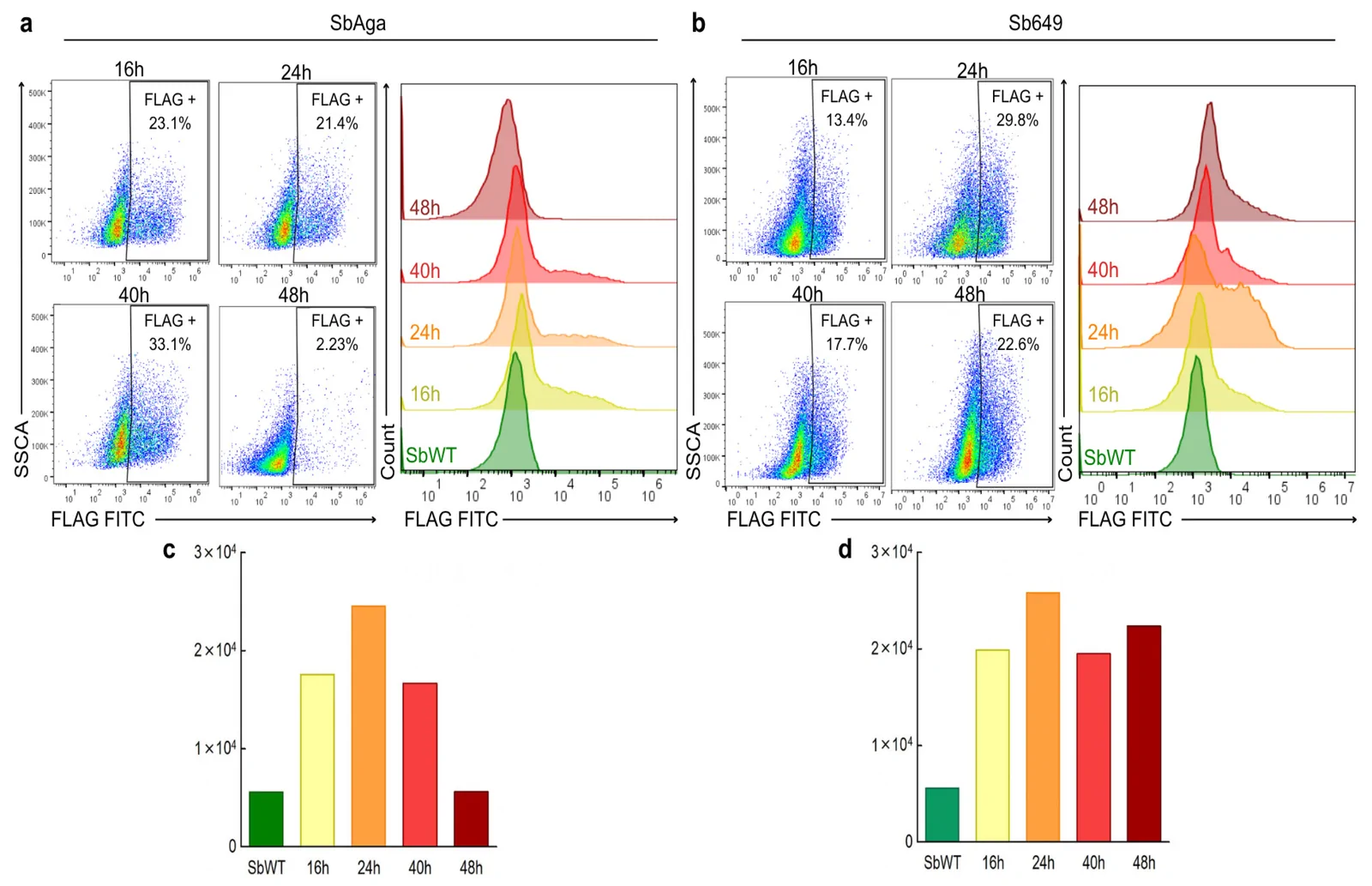
Representative flow cytometry analysis of anti-CD3 scFv surface display by *Saccharomyces boulardii* during cultivation. (**a**) SbAga and (**b**) Sb649 strains cultivated for 16, 24, 40, or 48 h at 30 °C and 200 rpm. Surface display was assessed through detection of the FLAG tag using a rabbit anti-FLAG antibody followed by FITC-conjugated anti-rabbit IgG. The percentage of FLAG-positive cells is indicated in each dot plot. Histograms show the fluorescence distribution at each cultivation time point, with SbWT included as a negative control. (**c**,**d**) Median fluorescence intensity (MFI) of the FLAG-positive population for SbAga and Sb649, respectively, at each cultivation time point. The gating strategy and additional negative controls are shown in Supplementary Figure S1.

Flow cytometry was used to assess the surface display of the anti-CD3 scFv on the yeast cell wall through detection of its FLAG tag at different time points during cultivation (Figure 2). *S. boulardii* (SbWT) labeled with Anti-Flag FLAG and Anti-IgG FITC, and as well as transformants Sb649 and SbAga labeled with only the secondary antibody, and unstained samples served as negative controls (Supplementary Figure S1). In the representative profiles, SbAga (Figure 2a) showed the highest proportion of FLAG-positive cells at 40 h, followed by a decline at 48 h, while Sb649 (Figure 2b) showed the highest proportion of FLAG-positive cells at 24 h and maintained a higher proportion of FLAG-positive cells at 48 h. Median fluorescence intensity (MFI) values for the FLAG-positive population are shown in Figure 2c,d for SbAga and Sb649, respectively, providing an additional quantitative measure of FLAG-associated fluorescence at each cultivation time point.

### 2.2. Effect of Oral Administration of Sb649 on DSS-Induced Colitis

**Figure 3:**
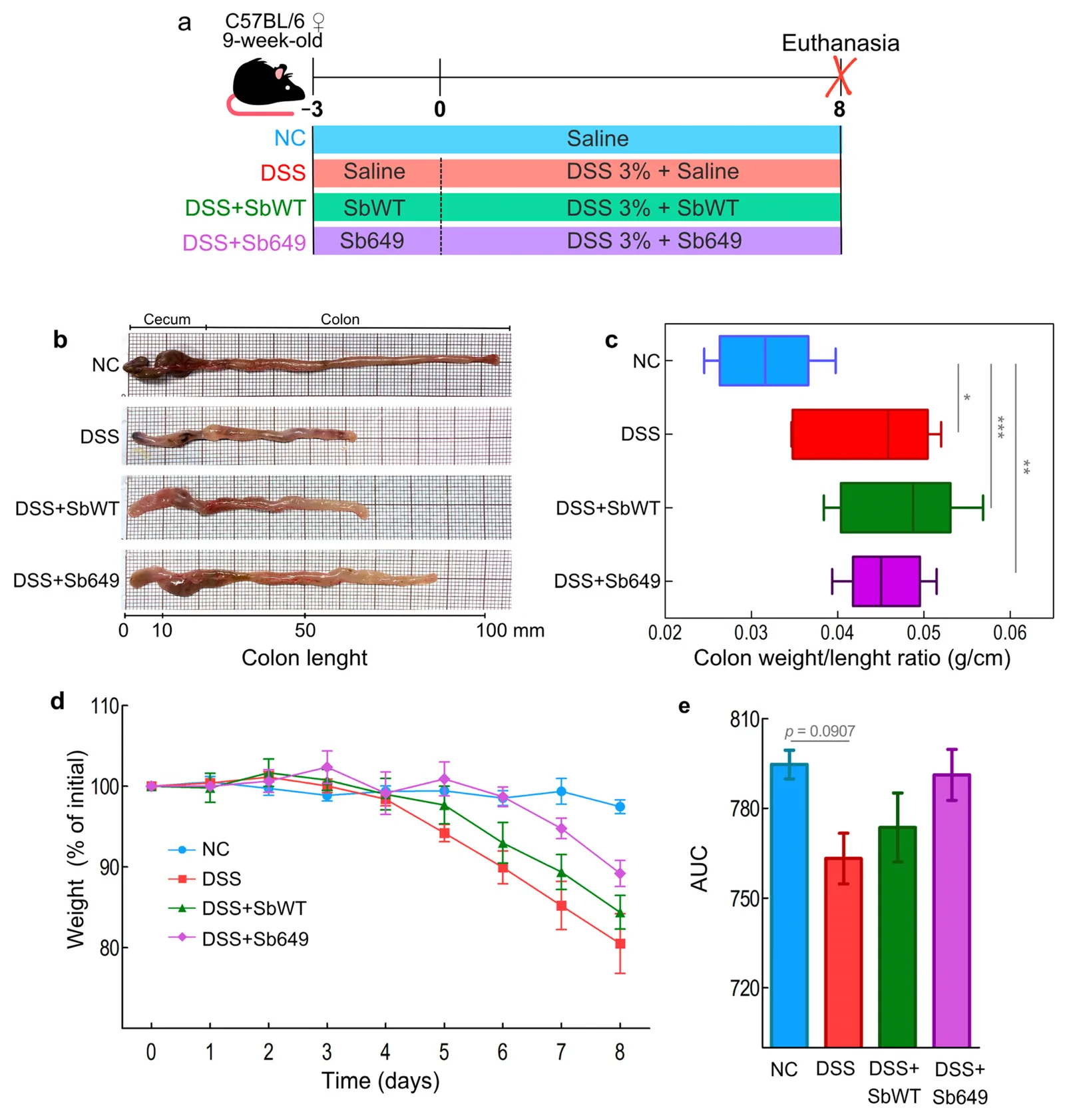
Experimental design of the DSS-induced colitis model and analysis of intestinal parameters and body weight. (**a**) Experimental design of the DSS-induced colitis model. Colitis was induced on day 0 by administering 3% DSS in filtered drinking water *ad libitum* until the end of the experiment. Mice were euthanized on day 8. Experimental groups included healthy control (NC), untreated colitis (DSS), colitis treated with wild-type *S. boulardii* (DSS+SbWT), and colitis treated with anti-CD3-displaying *S. boulardii* (DSS+Sb649). (**b**) Representative colon samples from each group. (**c**) Colon weight-to-length ratio. (**d**) Body weight change (%) throughout the experiment relative to day 0, when colitis induction began. The grey arrow indicates the final experimental day (day 8), which is shown separately in (**e**). Animals were weighed daily. Data are presented as mean ± SEM. Longitudinal body weight changes (**d**) were analyzed using a linear mixed-effects model, with group, day, and their interaction as fixed effects and animal as a random effect. Statistical analyses were performed using one-way ANOVA followed by Tukey’s multiple comparisons test for colon weight-to-length ratio (**c**) and the Kruskal–Wallis test followed by Dunn’s multiple comparisons test for body weight on day 8 (**e**). Statistical significance is indicated as * for *p* < 0.05, ** for *p* < 0.01, and *** for *p* < 0.001.

To evaluate *S. boulardii* as a delivery platform for recombinant antibodies, we used a DSS-induced mouse model of colitis to assess the effects of oral administration of anti-CD3 scFv-displaying yeast on disease progression (Figure 3a). Sb649 was chosen for gavage in mice due to its greater expression stability.

Disease induction resulted in colon shortening in the diseased groups (Figure 3b) and an increased colon weight-to-length ratio compared with the healthy control (NC) (Figure 3c). However, no significant differences were observed between the yeast-treated groups and the untreated DSS group.

DSS-treated mice exhibited progressive body weight loss throughout the experiment (Figure 3d). Longitudinal analysis of body weight using a mixed-effects model revealed significant effects of group (*p* = 0.0073), day (*p* < 0.0001), and a significant group-by-day interaction (*p* < 0.0001), indicating distinct body weight trajectories among the experimental groups. At day 8, body weight was significantly reduced in the DSS and DSS+SbWT groups compared with the healthy control, whereas no significant difference was observed between the DSS+Sb649 and NC groups (Figure 3e).

**Figure 4:**
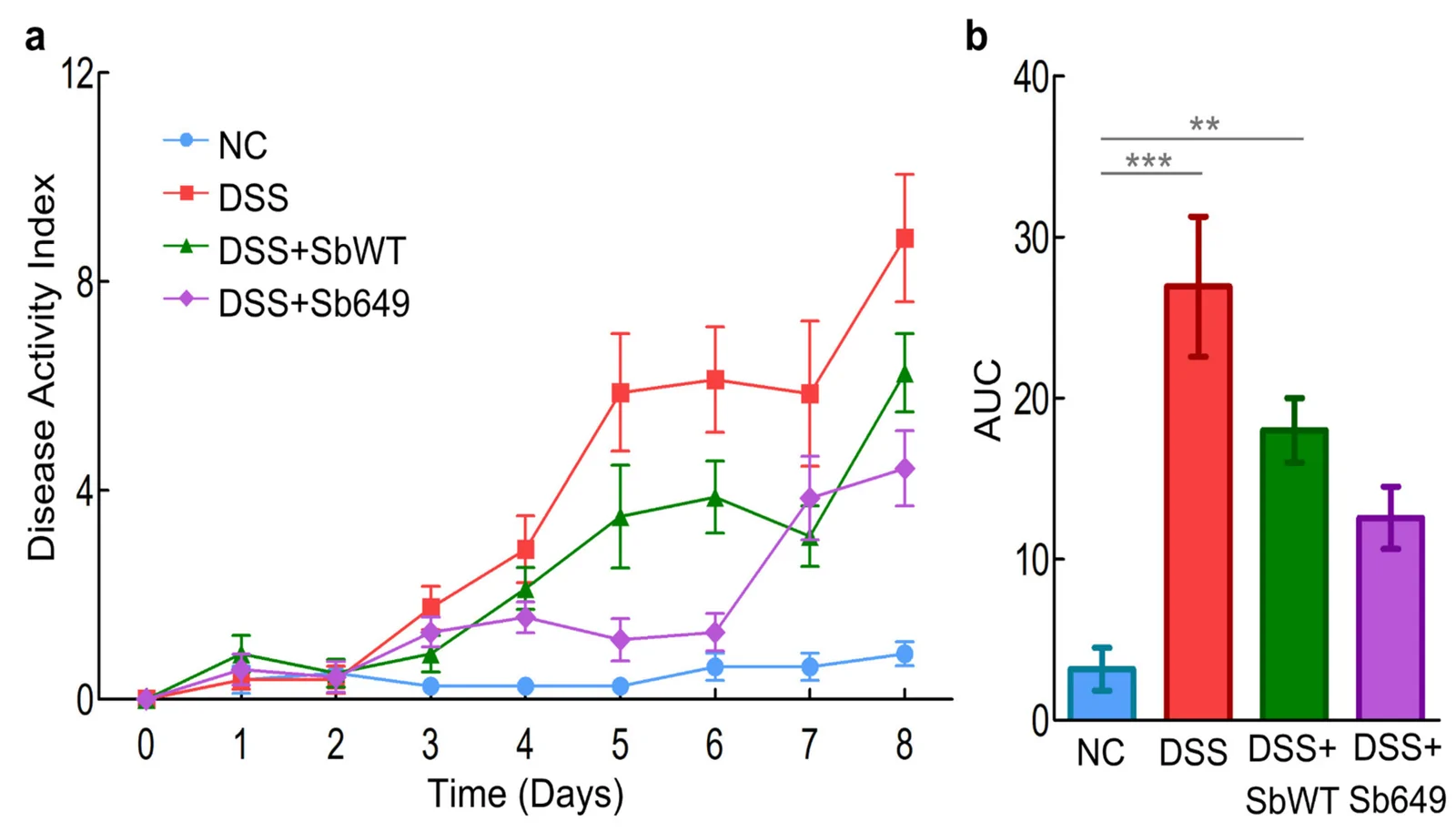
Analysis of disease activity progression. (**a**) Disease Activity Index (DAI) over time in the experimental groups, presented as mean ± SEM. (**b**) Area under the curve (AUC) of DAI. Experimental groups included healthy control (NC), untreated colitis (DSS), colitis treated with wild-type *S. boulardii* (DSS+SbWT), and colitis treated with anti-CD3-displaying *S. boulardii* (DSS+Sb649). Longitudinal DAI values (**a**) were analyzed using a linear mixed-effects model, with group, day, and their interaction as fixed effects and animal as a random effect. DAI AUC (**b**) was analyzed using the Kruskal–Wallis test followed by Dunn’s multiple comparisons test. Statistical significance is indicated by ** for *p* < 0.01 and *** for *p* < 0.001.

Weight loss, rectal bleeding, and stool consistency were used to calculate the disease activity index (DAI), as described by Cooper and colleagues (1993). DAI was calculated daily for each group to monitor changes during the experiment (Figure 4a). Longitudinal analysis of DAI using a mixed-effects model revealed significant effects of group, day, and a significant group-by-day interaction (*p* < 0.0001 for all). Both SbWT and Sb649 showed significantly lower DAI values than untreated DSS mice from day 5 to day 8. Overall, the group that received Sb649 maintained lower DAI values throughout the experiment, with a more pronounced difference relative to untreated DSS mice on days 5, 6, and 8. Sb649 showed significantly lower DAI values than SbWT on days 5 and 6 (Supplementary Figure S2). Analysis of the area under the curve (AUC) showed significantly higher cumulative disease activity in the DSS and DSS+SbWT groups than in the NC group, with no statistical differences between NC and DSS+Sb649 groups (Figure 4b).

### 2.3. Modulation of Genes Involved in Immune Responses

**Figure 5:**
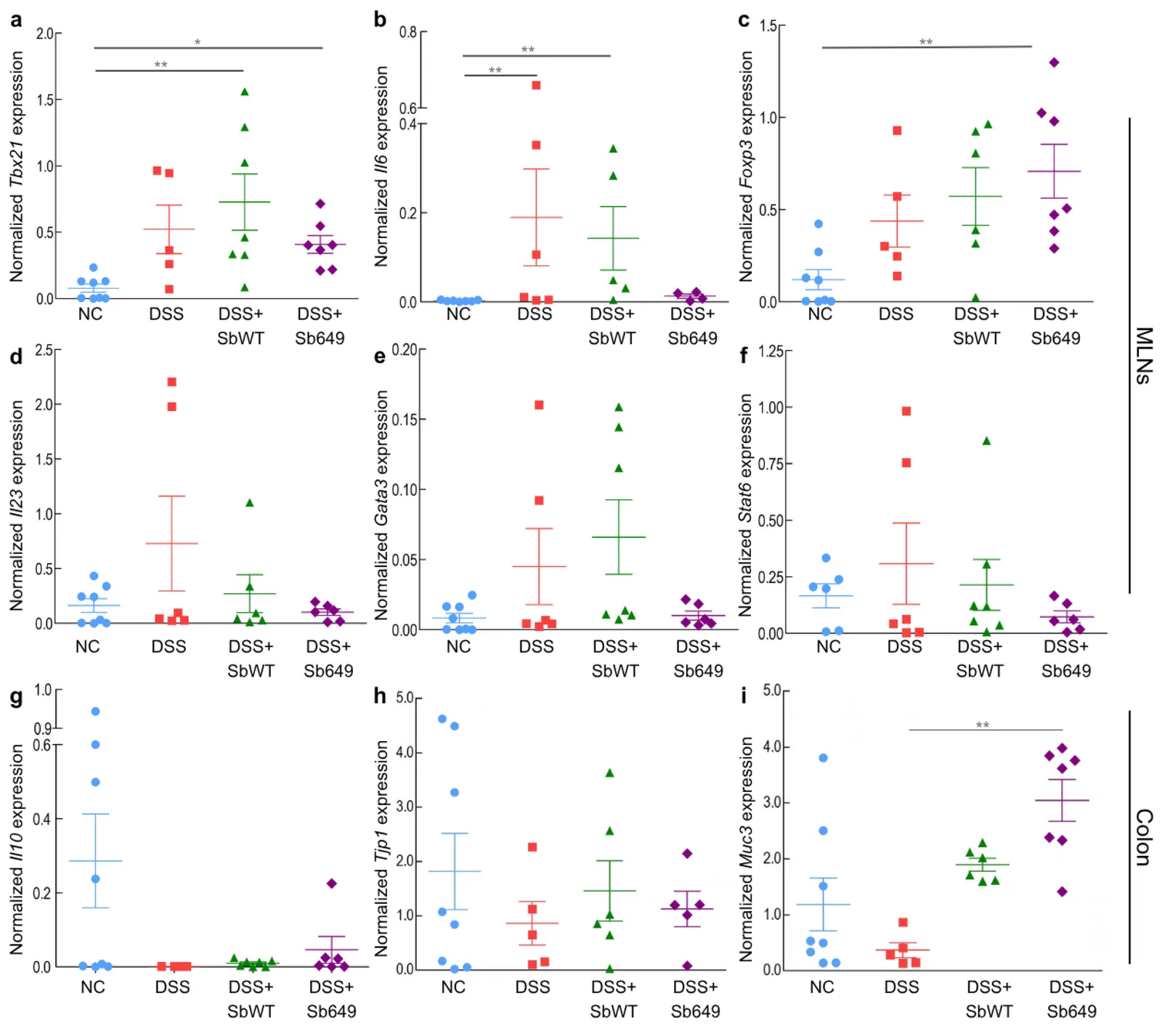
Expression of inflammation-related genes in mesenteric lymph nodes (MLNs) and colon tissue determined by qPCR. Tissues were collected from healthy control (NC), untreated colitis (DSS), colitis treated with wild-type *S. boulardii* (DSS+SbWT), and colitis treated with anti-CD3-displaying *S. boulardii* (DSS+Sb649) mice. Expression levels of (**a**) *Tbx21*, (**b**) *Il6*, (**c**) *Foxp3*, (**d**) *Il23*, (**e**) *Gata3*, and (**f**) *Stat6* in MLNs were normalized to the endogenous control *Actb*. Expression levels of (**g**) *Il10*, (**h**) *Tjp1*, and (**i**) *Muc3* in colon tissue were normalized to the endogenous control *B2m*. Outliers were identified using the interquartile range (IQR) method and excluded. The number of excluded observations and the effect of their exclusion on statistical significance are reported in Supplementary Table S1. Statistical analysis was performed using the Kruskal–Wallis test followed by Dunn’s multiple comparisons test. Statistical significance is indicated as * for *p* < 0.05 and ** for *p* < 0.01.

Exploratory analysis on gene expression in mesenteric lymph nodes (MLNs) and colon cells was carried out to characterize the response to oral administration of yeast displaying anti-CD3 scFv (Figure 5).

In MLNs, *Tbx21* expression was significantly increased in the yeast-treated diseased groups (SbWT or Sb649) compared to the NC group (Figure 5a). *Il6* was also significantly upregulated in the DSS and DSS+SbWT groups, whereas the DSS+Sb649 group showed no significant increase (Figure 5b). In contrast, *Foxp3* expression increased significantly only in the DSS+Sb649 group compared with the NC group, but did not differ significantly among the DSS-treated groups (Figure 5c). Numerically higher expression of the inflammation-related genes *Il23*, *Gata3* and *Stat6* was observed in the DSS and DSS+SbWT groups, but not in the DSS+Sb649 group. However, these changes were not statistically significant (Figure 5d–f). In the colon, *Il10* and *Tjp1* expression did not differ significantly among the groups (Figure 5g,h), but *Muc3* expression was significantly higher in DSS+Sb649 mice than in the DSS group (Figure 5i).

## 3. Discussion

This study explored the potential of *S. boulardii* as an oral vector for anti-CD3 scFv and its effects on DSS-induced colitis. The ability of *S. boulardii* to perform eukaryotic post-translational modifications makes it a suitable host for production, folding, and surface display of complex proteins, including antibody fragments [19]. This approach was recently demonstrated with an anti-TNFα scFv, which reduced inflammation in a murine colitis model [20].

To improve antibody accessibility, two plasmids containing different stalk sequences (AGA_Stalk and 649_Stalk) were constructed. In both constructs, the SED1 signal peptide and anchoring domain directed the scFv to the cell wall, and the *SED1* promoter drove expression. The *SED1* gene encodes a cell wall glycoprotein that is constitutively expressed, particularly in the stationary phase and further induced under stress conditions such as high cell density, osmotic stress, and nutrient limitation [21,22]. These stress responses are relevant in vivo, as the GIT presents environmental challenges, including low pH, limited oxygen and nutrients, and host body temperature, which can affect microbial survival and microbiota composition [23,24].

Sb649 was selected for the in vivo experiment based on the representative flow cytometry profiles, in which it maintained a higher proportion of FLAG-positive cells at 48 h than SbAga. The surface detection of the FLAG epitope suggests the display of the associated anti-CD3 scFv. The DSS-induced colitis model was chosen here because it is widely used to study UC, in which CD4+ T-cells play a central role in maintaining chronic inflammation [4]. Oral anti-CD3 therapy can ameliorate UC symptoms in both clinical studies and murine models by targeting T lymphocytes [6,25].

DSS-treated mice developed rectal bleeding, weight loss, and diarrhea, severe inflammatory symptoms that are consistent with disease induction [26]. It has been observed that *S. boulardii* reduces symptoms and disease activity in animal models and contributes to remission in humans when combined with mesalazine [27,28]. In the present study, the longitudinal analysis showed that administration of both SbWT and Sb649 had significantly lower DAI values than untreated DSS mice from day 5 to day 8, with Sb649-treated mice also showing significantly lower DAI values than SbWT-treated mice on days 5 and 6. In the cumulative analysis, DAI AUC was significantly higher in the DSS and DSS+SbWT groups than in the healthy control group, whereas the DSS+Sb649 group did not differ significantly from the healthy control. However, no significant differences in DAI AUC were observed among the DSS-treated groups. This pattern may indicate a modest protective effect of Sb649 during the establishment of acute DSS-induced colitis and suggests that the surface-displayed anti-CD3 scFv may have contributed to the additional effects observed with Sb649 administration.

Anti-CD3 therapy is known to induce tolerance at the cellular level by modulating the expression of regulatory genes [29]. Therefore, we conducted an exploratory analysis to investigate whether yeast displaying anti-CD3 scFv was associated with changes in gene expression in mesenteric lymph nodes and colon tissue, two important sites at the interface between the gut and the immune system that contribute to the establishment of immune tolerance [30,31].

In MLNs, dendritic cells migrating from the intestine present antigens and drive T-cell differentiation and proliferation [32]. In this tissue, *Il6* expression was significantly increased in the DSS and DSS+SbWT groups compared with the NC group, whereas DSS+Sb649 did not show a significant increase. IL-6 is a key mediator of chronic intestinal inflammation because it promotes pro-inflammatory cytokine production, T-cell activation and T-cell activity by preventing apoptosis [33]. This gene is highly expressed in DSS-induced colitis compared with healthy animals, supporting its involvement in disease pathogenesis [34].

The binding of anti-CD3 can induce T-cell anergy and promote immunoregulatory mechanisms that can help control exacerbated inflammation [2]. This mechanism could explain the comparatively lower *Il6* expression in DSS+Sb649, as well as the delayed disease progression. However, *Il6* expression did not differ significantly among all DSS-treated groups. Therefore, this should be considered a mechanistic hypothesis to be confirmed by further investigations.

*Foxp3* expression was significantly increased only in the DSS+Sb649 group compared with the healthy control. Foxp3 is a marker of the Treg profile and regulates the development and function of these cells [35]. Although Foxp3 expression did not differ significantly between Sb649 and the other DSS-treated groups, the significant increase in the DSS+Sb649 group may indicate enhanced activation of regulatory pathways under this experimental condition. However, additional cellular analyses are required to determine whether this transcriptional pattern reflects changes in regulatory T-cell populations.

T-bet, encoded by *Tbx21*, is an important regulatory factor in Th1 differentiation with a key role in mucosal T-cell activation and intestinal inflammation [36]. *Tbx21* expression increased after DSS exposure, but the difference relative to the healthy control was statistically significant only for the DSS+SbWT and the DSS+Sb649 groups. Dendritic cells exposed to *S. boulardii* have been reported to induce IFN-γ production when co-incubated with naive T-cells, suggesting a shift toward a Th1 profile [37]. This may explain why Tbx21 expression remained elevated in both yeast-treated groups. However, neither yeast-treated group differed significantly from the untreated DSS group. Therefore, the increased Tbx21 expression cannot be attributed specifically to yeast administration and may, at least in part, reflect the inflammatory environment induced by DSS. Despite this result, the significant increase in *Il6* observed in the DSS and DSS+SbWT groups relative to NC was not observed in the DSS+Sb649 group, while *Foxp3* expression was significantly increased relative to NC. This suggests a complex transcriptional response associated with Sb649 treatment, potentially involving regulatory mechanisms that limit excessive immune responses rather than directly suppressing them. However, the contribution of the surface-displayed anti-CD3 scFv to this response needs to be further explored.

The colonic epithelium is directly affected by DSS administration, resulting in tissue injury and disruption of barrier integrity [38]. Therefore, the expression of genes associated with mucosal protection and epithelial integrity was also evaluated in the colon. At the immunoregulatory level, no significant difference was observed in *Il10* expression, a cytokine associated with the Treg profile. Likewise, *Tjp1*, which encodes structural proteins involved in epithelial barrier integrity, also showed no significant change. Because animals were euthanized during the acute phase of colitis, ongoing epithelial injury may have limited detection of the treatment’s impact on genes related to epithelial maintenance and repair.

In contrast, *Muc3* expression was significantly increased in the DSS+Sb649 group compared with the DSS group. Reduced mucin expression is observed during DSS-induced colitis, and administration of *S. boulardii* preserved mucus production and improved epithelial barrier function [15,39]. The increased *Muc3* expression in DSS+Sb649 compared with untreated DSS mice may therefore reflect changes in mucosal responses associated with Sb649 treatment. One possible explanation is that local immune modulation associated with anti-CD3 exposure may alter the cytokine landscape and immune–epithelial interactions during DSS-induced injury, indirectly influencing mucin expression and epithelial barrier responses.

Although the observed effects were modest, Sb649 administration was associated with a distinct clinical and transcriptional pattern related to a less inflammatory profile. Despite that, some limitations should be considered. First, surface display was assessed by detection of the FLAG tag fused to the recombinant scFv. Therefore, these experiments demonstrate FLAG-tag accessibility on the yeast surface but do not establish whether the displayed scFv retains its native conformation or CD3-binding activity. Direct binding or functional assays will be required to confirm the biological activity of the surface-displayed scFv. Also, only a fraction of the yeast population displayed anti-CD3 scFv on its surface, and the amount of antibody fragments reaching the colon and their biological activity in vivo remain uncertain. In addition, histopathological assessment was not performed in this study, limiting the evaluation of tissue injury and inflammation independently of the clinical and molecular parameters. Moreover, although the transcriptional profile observed in MLNs suggests modulation of immune responses, characterizing immune cell populations is necessary to determine whether the observed changes reflect alterations in lymphocyte subsets or gene expression within specific cell populations. Finally, the experimental design employed here may have underestimated the combined effect of *S. boulardii* and recombinant anti-CD3 due to DSS-induced acute toxicity and extensive epithelial injury. Since oral anti-CD3 primarily targets T-cell responses, chronic or cyclic DSS models [40] may provide a more suitable platform to evaluate the full therapeutic potential of recombinant *S. boulardii* expressing anti-CD3.

## 4. Materials and Methods

### 4.1. Strains and Growth Conditions

XL1-Blue *Escherichia coli* (Stratagene, La Jolla, CA, USA) was used to amplify plasmids. Cultures were grown in Luria-Bertani (LB) (1% casein peptone, 0.5% yeast extract, 1% NaCl, pH 7.0) at 37 °C and 250 rpm. *Saccharomyces boulardii* 17 yeast was isolated from REPOFLOR^®^ tablets (EMS, São Paulo, Brazil, Lot: L2 × 7916) by plating on yeast peptone dextrose (YPD) agar (2% casein peptone, 1% yeast extract, 2% glucose, and 1.5% agar), and plates were incubated at 30 °C for 48 h. For liquid cultures, yeast cells were grown in liquid YPD (2% casein peptone, 1% yeast extract, and 2% glucose). Cultures were maintained at 200 rpm and 30 °C. Transformants were selected on YPD supplemented with 200 μg/mL geneticin (Thermo Fisher Scientific, Waltham, MA, USA). In this study, the wild-type yeast is referred to as SbWT, and the transformant strains are designated Sb649 and SbAga according to their stalk domains.

### 4.2. Plasmids

The vector used in this work was derived from pYC230 [41]. As a shuttle vector, it contains the *ColE1* origin of replication and ampicillin resistance gene (*AmpR*) for clonal expansion and transformant selection in *Escherichia coli* and a 2µ origin for independent plasmid replication in yeast [42], and the geneticin resistance gene (*G418R*) as a selective eukaryotic marker. The plasmids were engineered to contain, between the Pst I and Not I sites, the SED1 promoter and anchoring region for efficient cell-surface display of heterologous proteins in *Saccharomyces cerevisiae* [21,43]. The *SED1* promoter includes 800 bp upstream of the ORF start codon to preserve native regulatory elements. This promoter drives the expression of a fusion protein containing the scFv fragment derived from the mouse anti-CD3 antibody sequence (clone 145-2C11) [44]. The SED1 leader peptide (GenBank: NC_001136.10, 599993..600849) was employed to direct the heterologous protein to the cell surface. Hemagglutinin (HA) and FLAG tag sequences were added to the amino and carboxy termini of the scFv, respectively, for specific labeling, followed by a GSGS (*GS2*) spacer sequence. Two different stalk sequences were tested. The AGA_Stalk sequence was designed based on the Aga1 of *S. cerevisiae* (NM_001183221) from position 304 to 1167. The codons for Cys at positions 316–318 were substituted with Ala codons. 649_Stalk was previously described [45]. The difference in stalk length was used to evaluate the impact of the antibody fragment’s projection from the cell surface. The carboxy-terminus of *SED1* (GenBank: X66838, 672..1358) was placed at the end of the fusion ORF. The transcriptional terminator of the *S. cerevisiae PGK1* gene completes the expression cassette (GenBank: NC_001135.5, 138997..139274).

### 4.3. Electroporation of S. boulardii

After growth on YPD agar, isolated colonies were inoculated into 5 mL of liquid YPD and incubated for 24 h. Subsequently, 1 mL of the pre-inoculum was transferred to 100 mL YPD and incubated at 30 °C and 200 rpm until the optical density (OD_600_) reached 1.6. Cells were harvested, washed with sterile distilled water and resuspended in 1 mL aliquots of 2 M sorbitol and 0.1 M CaCl_2_, then stored at −80 °C until use. Before electroporation, aliquots were thawed at room temperature, washed once with distilled water, and twice with 2 M sorbitol. Cells were resuspended in 400 μL of 2 M sorbitol and incubated with 20 μg of plasmid DNA and 10 μg of herring sperm carrier DNA on ice for 5 min. The mixture was transferred to ice-cold 0.2 cm electroporation cuvettes (Sigma-Aldrich, St. Louis, MO, USA) and pulsed at 2.5 kV, 25 μF, and 200 Ω using GenePulser Xcell (Bio-Rad, Hercules, CA, USA). Immediately after the pulse, cells were resuspended in 1 mL of 2 M sorbitol and incubated at 30 °C for 2 h. Later, 1 mL of YPD was added, and incubation continued for one hour. Cells were then centrifuged, resuspended in 200 μL of YPD, and plated onto selective YPD agar plates using sterile glass beads. After 48 h, transformant colonies were collected for subsequent tests.

### 4.4. Flow Cytometry Assays

Flow cytometry analyses were performed using an Accuri C6 Plus flow cytometer (Becton, Dickinson and Company, Franklin Lakes, NJ, USA). Data were analyzed using FlowJo software, version 10.10 (FlowJo, LLC, Ashland, OR, USA, RRID: SCR_008520). To assess surface display of anti-CD3 scFv, 1 mL of each culture was centrifuged and washed three times with phosphate-buffered saline (PBS). Ten μL of the cell suspension was incubated with 3 μL of rabbit anti-FLAG antibody (Sigma-Aldrich Cat# F2555, RRID: AB_796202) in a final volume of 30 μL for 1 h on ice. Samples were then washed with PBS and resuspended in 30 μL for incubation with anti-rabbit IgG–FITC (Sigma-Aldrich Cat# F0382, RRID: AB_259384) for 30 min on ice. After three washes with 1 mL of PBS, cells were resuspended to a final volume of 100 μL for acquisition.

The gating strategy used for flow cytometry analysis is described in Supplementary Figure S1. The FLAG-positive population was defined based on unstained cells, cells incubated with the secondary antibody only, and SbWT stained with anti-FLAG followed by anti-rabbit IgG–FITC. These controls were used to establish the fluorescence threshold for FLAG-positive cells, which was then applied to samples collected at the different cultivation time points. Median fluorescence intensity (MFI) was determined within the FLAG-positive population at each cultivation time point.

### 4.5. In Vivo Experimental Design and Dose Preparation

Nine-week-old female C57BL/6 mice were obtained from the Multidisciplinary Center for Biological Research (CEMIB) at the State University of Campinas, São Paulo, Brazil. All experimental procedures were approved by the Ethics Committee on Animal Use (CEUA) of the University of Brasília (protocol 78/2018) and performed in accordance with guidelines and regulations. Animals were housed under controlled conditions with a 12-h light-dark cycle at room temperature. Before the experiment, mice were cohoused for three weeks by random assignment to cages (four animals per cage).

A total of 32 mice were initially allocated to the four experimental groups (n = 8 per group). One mouse from the DSS+Sb649 group died before DSS induction and therefore was not included in longitudinal analyses. During induction, two mice from the DSS group died before the final time point, resulting in missing body weight and DAI measurements at subsequent time points.

Mice received daily oral gavage of SbWT, Sb649, or 0.9% saline starting on day- −3. Acute colitis was induced on day 0 by administration of 3% (*w*/*v*) dextran sulfate sodium (DSS; MW 36,000–50,000 Da; MP Biomedicals, Santa Ana, CA, USA) in filtered drinking water *ad libitum*. Daily liquid intake was recorded (mean 2.75 mL per animal). On day 8, mice were euthanized by intraperitoneal administration of thiopental (200 mg/kg). Mesenteric lymph nodes and colon samples from each animal were collected for RNA extraction, and colons were weighed and measured.

For dose preparation, isolated colonies were inoculated into 5 mL YPD (30 °C, 200 rpm, 24 h) and used to inoculate 100 mL of YPD at an OD_600_ of 0.2. Sb649 was grown in selective YPD. The number of colony forming units (CFU) per dose was determined from freshly grown cultures by serial dilution (10^6^–10^8^) and plating with sterile glass beads. Colonies were counted after 48 h of incubation, resulting in 9 × 10^8^ to 1.8 × 10^9^ CFU per dose. Before administration, cultures were centrifuged, washed and resuspended in saline. The cell suspension was mixed with 25% glycerol to a final volume of 1 mL and stored at −80 °C. After thawing, cell viability was assessed by propidium iodide (PI) staining and surface display by anti-FLAG staining followed by anti-rabbit IgG–FITC (Supplementary Figure S3).

### 4.6. Analysis of Macroscopic Parameters

Throughout the experiment, animals were weighed, and fecal samples were collected daily. Disease activity index (DAI) was determined based on body weight loss, stool consistency, and rectal bleeding [46]. Body weight loss was calculated daily as the percentage change relative to body weight on day 0, before DSS administration, and scored as: 0 (no loss), 1 (1–5%), 2 (6–10%), 3 (11–20%), and 4 (>20%). Rectal bleeding was scored as 0 (none), 1 (mild), 2 (moderate), 3 (severe), and 4 (very severe). Stool consistency was scored as 0 (normal), 1 (moderately loose), 2 (very loose), 3 (diarrhea), and 4 (liquid diarrhea). In addition to DAI parameters, the colon weight-to-length ratio was also analyzed.

### 4.7. Gene Expression

After euthanasia, colons and mesenteric lymph nodes were collected, immersed in QIAzol Lysis Reagent (Qiagen, Valencia, CA, USA), and stored at −80 °C. Tissues were homogenized with 5 mm tungsten carbide beads in TissueLyser LT (Qiagen, Valencia, CA, USA). Total RNA was extracted with the RNeasy Mini Kit (Qiagen, Valencia, CA, USA) according to the manufacturer’s instructions. For quantitative PCR (qPCR), primers targeting immune markers, cell activation, and epithelial barrier integrity were used (Supplementary Table S2). Gene expression was normalized to the endogenous controls beta-actin (*Actb*) for lymph nodes and beta-2-microglobulin (*B2m*) for colon samples.

### 4.8. Statistical Analyses

Statistical analyses and graphs were performed using GraphPad Prism version 5.0 (GraphPad Software, La Jolla, CA, USA, RRID: SCR_002798) and R version 4.6.0 (R Foundation for Statistical Computing, Vienna, Austria). Data are presented as mean ± standard error of the mean (SEM). Each animal was considered an experimental unit. Colon weight-to-length ratio was analyzed using one-way analysis of variance (ANOVA) followed by Tukey’s multiple comparisons test. Longitudinal body weight changes (%) were analyzed using a linear mixed-effects model, with group, day, and their interaction as fixed effects and animal as a random effect. Body weight change at day 8 was additionally analyzed as an endpoint measure with Kruskal–Wallis test followed by Dunn’s multiple comparisons test. DAI was analyzed using the same linear mixed-effects approach, and post-hoc pairwise comparisons among groups at individual time points were performed using estimated marginal means (emmeans) with Tukey’s adjustment for multiple comparisons. DAI AUC was calculated to summarize cumulative disease activity over the experimental period and analyzed using the Kruskal–Wallis test followed by Dunn’s multiple comparisons test. Gene expression data were analyzed using the Kruskal–Wallis test followed by Dunn’s multiple comparisons test. qPCR analyses were considered exploratory. Outliers were identified using the interquartile range (IQR) method and excluded. The number of excluded observations and the effect of their exclusion on statistical significance are reported in Supplementary Table S1. Differences were considered statistically significant at *p* < 0.05.

## 5. Conclusions

In this study, we demonstrated that *S. boulardii* can be genetically engineered for surface display of an anti-CD3 scFv on its surface. Two constructs with stalk domains of different lengths were created to evaluate antibody accessibility, and the strain with the longer stalk, Sb649, showed more stable surface display during cultivation. In vivo, longitudinal analysis showed significantly lower DAI values in Sb649-treated mice than in untreated DSS mice from day 5 to day 8 and then in SbWT-treated mice on days 5 and 6. Sb649 treatment was also associated with a distinct transcriptional pattern, with increased Foxp3 expression relative to the healthy control and increased Muc3 expression relative to DSS-treated mice. Together, these findings suggest a potential protective effect of Sb649 during DSS-induced colitis, although further studies are necessary to optimize this strategy and clarify its effects on the inflammatory environment.

## Data Availability

The original contributions presented in this study are included in the article/Supplementary Materials. Further inquiries can be directed to the corresponding author.

## Supplementary Materials

The following supporting information can be downloaded at: https://www.mdpi.com/article/10.3390/ijms27208959/s1.
