# Supplementary material for: Surface Display of an Anti-CD3 scFv by Saccharomyces boulardii and Its Effect on DSS-Induced Colitis in Mice
Source: Int J Mol Sci. 2026 Oct 9;27(20):8959. doi: 10.3390/ijms27208959 (PMC13704555; doi:10.3390/ijms27208959)
Supplement: Supplementary file 1 [file ijms-27-08959-s001.zip › ijms-4586428-supplementary.pdf]

Supplementary Material

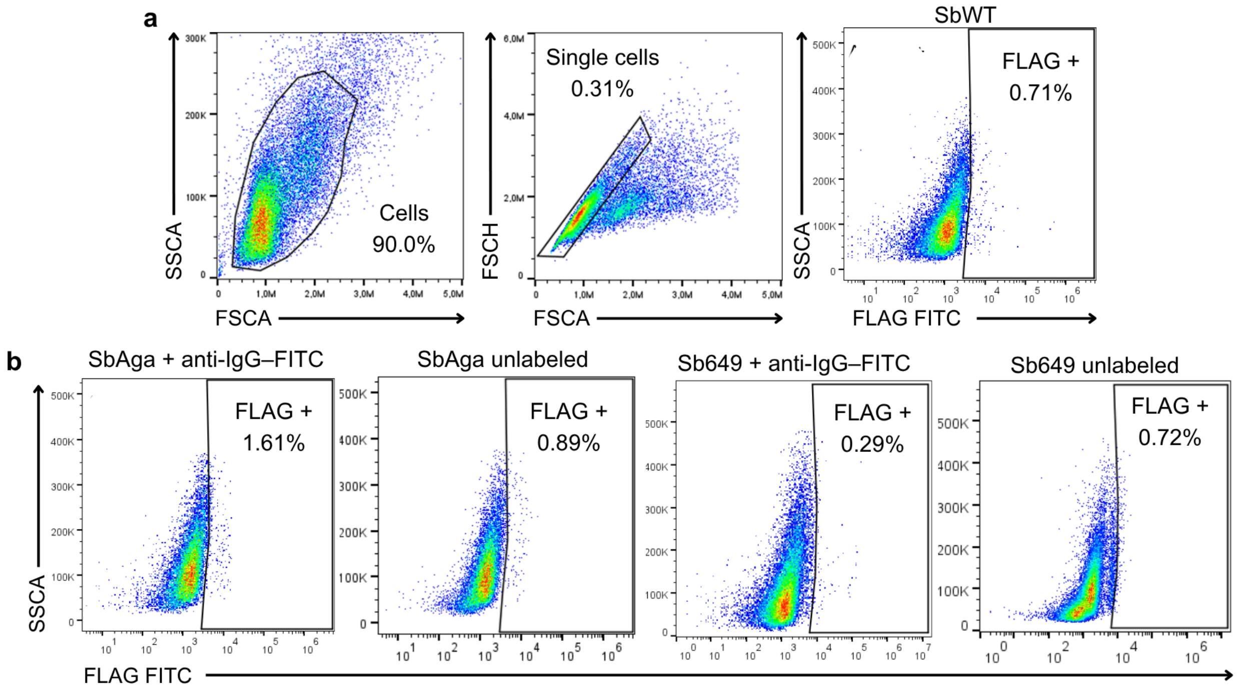

Supplementary Figure S1. Gating strategy and flow cytometry controls. **(a)** The gating strategy consisted of sequentially identifying the homogeneous cell population based on FSC/SSC parameters, selecting single cells, and subsequently gating the FLAG-positive population. Representative gating is shown for SbWT stained with anti-FLAG and the FITC-conjugated anti-rabbit IgG. **(b)** Flow cytometry controls used to establish the FLAG-positive threshold, including SbAga and Sb649 transformants stained with only the FITC-conjugated anti-rabbit IgG, as well as the corresponding unlabeled controls.

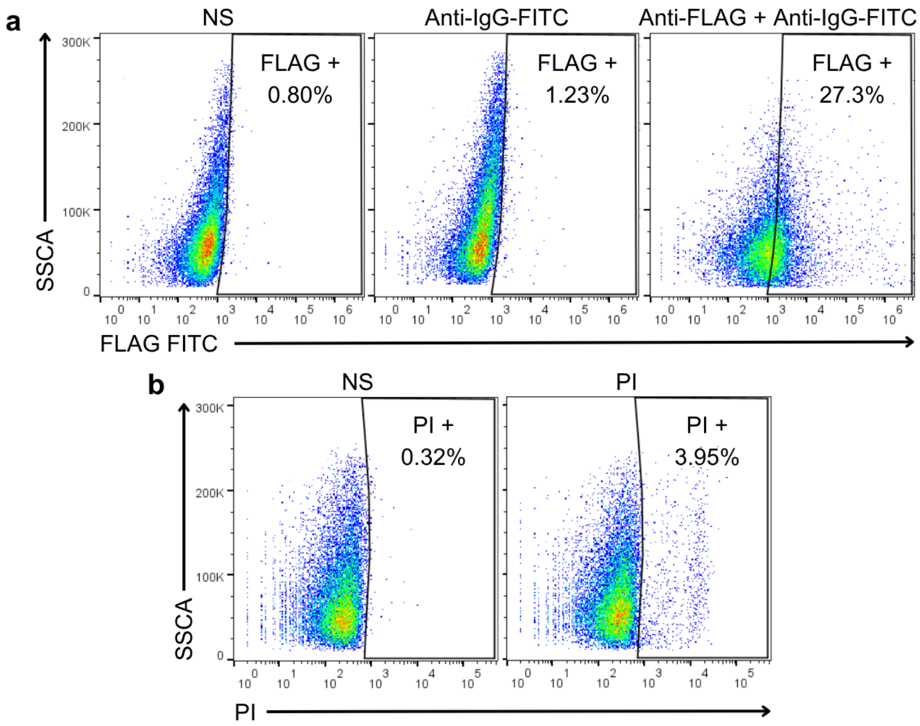

Supplementary Figure S2. Viability and surface display of Sb649 after thawing of the prepared doses. **(a)** Representative flow cytometry profiles of Sb649 after thawing. Surface display was assessed by anti-FLAG staining followed by anti-rabbit IgG–FITC. Non-stained (NS) and secondary antibody-only (anti-IgG–FITC) samples were used as controls to define the FLAG-positive population. The percentage of FLAG-positive cells is indicated in each plot. **(b)** Cell viability after thawing was assessed by propidium iodide (PI) staining. Non-stained (NS) cells were used as the control to define the PI-positive population. The percentage of PI-positive cells is indicated in each plot.

Supplementary Table S1. Primers used for quantitative PCR tests of mice's mesenteric lymph nodes and colon

Gene	Sequence	Gene	Sequence
B2m	F: TGCTATCCAGAAAACCCCTCA	*ActB*	F: GCAGATGTGGATCAGCAAGC
	R: TTTCAATGTGAGGCGGGTGG		R: CAAAGCCATGCCAATGTTGT
Tbx21	F: TCAGGACTAGGCGAAGA	*Muc3*	F: CGTGGTCAACTGCGAGAATGG
	R: GCCTTCGGTTTCCTTATC		R: CGGCTCTATCTCTACGCTCTC
Il6	F: GCCTTCTTGGGACTGATGCT	*Tjp1*	F: GAGCGGGCTACCTTACTGAAC
	R: TGCCATTGCACAACTCTTTTCT		R: GTCATCTCTTTCCGAGGCATTAG
Il23	F: CAGCTCTCTCGGAATCTCTGC	*Stat6*	F: GGATTCAGATTTGGAAGCGGC
	R: GATCCTTTGCAAGCAGAACTGG		R: AATTTCCACCAGGCTTTCACAC
Foxp3	F: ACTCGCATGTTCGCCTACTT	*Il10*	F: GGTTGCCAAGCCTTATCGGA
	R: AGGGATTGGAGCACTTGTTG		R: GAGAAATCGATGACAGCGCC

Gata3	F: TTTACCCTCCGGCTTCATCCTCCT
	R: GCACCTGATACTTGAGGCACTCT

Supplementary Table S2. Outliers identified and excluded from the qPCR analysis using the interquartile range (IQR) method.

Tissue	Gene	Group(s) with exclusion	Number excluded	Significance changed?	All observations retained	After (significant pairs)
MLNs	*Tbx21*	DSS	1	Yes	NT vs DSS (*); NT vs DSS+SbWT (**)	NT vs DSS+SbWT (**); NT vs DSS+Sb649 (*)
MLNs	*Il6*	DSS+Sb649; NT	1; 1	Yes	None	NT vs DSS (*); NT vs DSS+SbWT (*)
MLNs	*Foxp3*	DSS+SbWT	2	Yes	NT vs DSS+SbWT (*); NT vs DSS+Sb649 (*)	NT vs DSS+Sb649 (**)
MLNs	*Il23*	DSS+SbWT; DSS+Sb649	1; 1	No	—	—
MLNs	*Gata3*	DSS+SbWT; DSS+Sb649	1; 1	No	—	—
MLNs	*Stat6*	DSS+SbWT	1	No	—	—
Colon	*Il10*	DSS+SbWT; DSS+Sb649	2; 1	No	—	—
Colon	*Tjp1*	DSS+SbWT; DSS+Sb649	2; 2	No	—	—
Colon	*Muc3*	DSS+SbWT	2	Yes	DSS vs DSS+SbWT (*); DSS vs DSS+Sb649 (**)	DSS vs DSS+Sb649 (**)

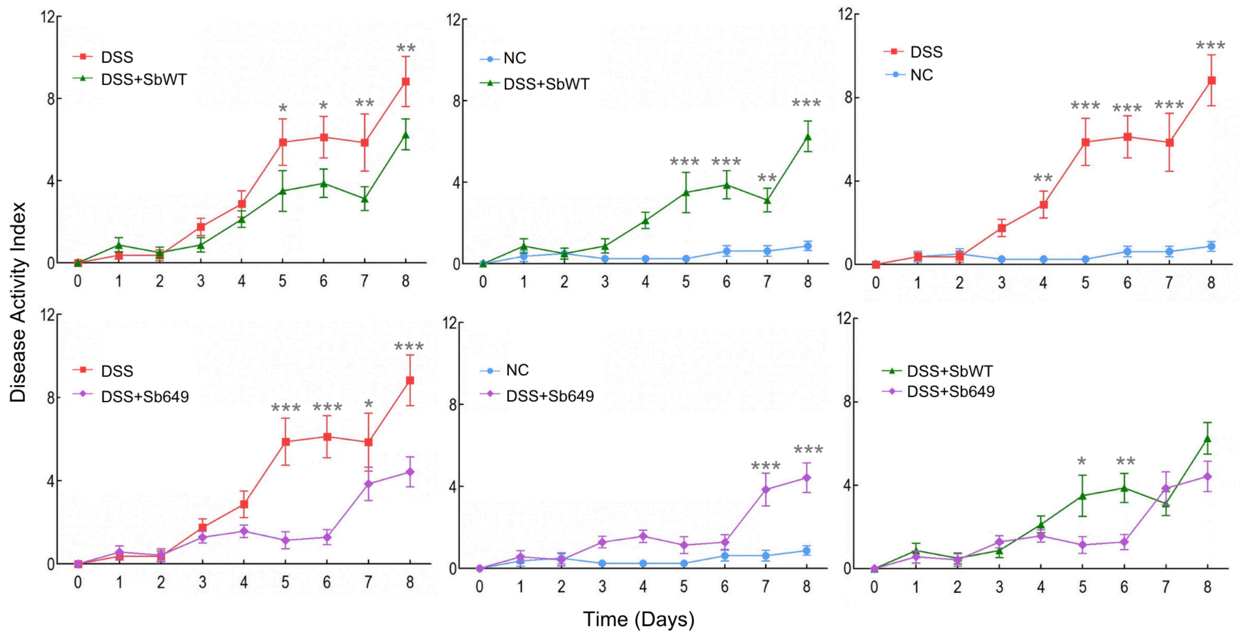

Supplementary Figure S3. Disease Activity Index (DAI) during DSS-induced colitis. Pairwise comparisons between NC, DSS, DSS+SbWT, and DSS+Sb649 groups are shown. Data are presented as mean ± SEM. Statistical analysis was performed using a linear mixed-effects model followed by Tukey-adjusted pairwise comparisons at each time point. Statistical significance is indicated as * for $p < 0.05$, ** for $p < 0.01$, and *** for $p < 0.001$.
